# Safety and Efficacy of Intraventricular Delivery of Bone Marrow-Derived Mesenchymal Stem Cells in Hemorrhagic Stroke Model

**DOI:** 10.1038/s41598-019-42182-1

**Published:** 2019-04-05

**Authors:** Peng Huang, William D. Freeman, Brandy H. Edenfield, Thomas G. Brott, James F. Meschia, Abba C. Zubair

**Affiliations:** 1 Department of Laboratory Medicine & Pathology, Mayo Clinic, Jacksonville, Florida USA; 2 Department of Neurology, Mayo Clinic, Jacksonville, Florida USA; 3 Department of Neurologic Surgery, Mayo Clinic, Jacksonville, Florida USA; 4 Department of Critical Care Medicine, Mayo Clinic, Jacksonville, Florida USA; 5 Cancer Basic Science Research, Mayo Clinic, Jacksonville, Florida USA

## Abstract

External ventricular drain (EVD) is used clinically to relieve intracranial pressure and occasionally to deliver medications following intracerebral hemorrhage (ICH). Mesenchymal stem cell (MSC) therapy has been shown to be neuroprotective and can induce neuroregeneration in stroke models. We evaluated the safety and efficacy of delivering MSCs intraventricularly in a rat hemorrhagic stroke model. Using autologous blood, hemorrhagic stroke was induced at specific coordinates in the right basal ganglia. After 30 minutes, rats were treated with either bone marrow-derived MSCs or a phosphate-buffered saline placebo via direct intraventricular infusion. Three dosages (2 × 10^5^/kg, 5 × 10^5^/kg, and 1 × 10^6^/kg) of MSCs were administered. Forelimb use asymmetry test was employed to evaluate functional improvement after cell therapy. At the end of the experiment, peripheral blood samples and organs were harvested; biochemistry, cytokine, and growth factor analysis and histology evaluations were performed to explore cell toxicity and cell fate, and the effects of MSC therapy on injury volume, anti-inflammation, and neurogenesis. Intraventricular administration of MSCs in ICH rat model showed improved behavior and alleviated brain damage. Additionally, treated ICH rats showed significantly reduced expression of IL-1α, IL-6, and IFN-γ. No obvious cell toxicity was noticed through blood chemistry and histology evaluation. None of the infused MSCs were detected at the end of the experiment. EVD is safe and effective to use as a method of delivering MSCs to treat ICH. Intraventricularly delivered MSCs have anti-inflammatory properties and a capacity to induce neurogenesis and improve function following ICH injury.

## Introduction

Intracerebral hemorrhage (ICH) is the least treatable form of stroke, causing high morbidity and mortality (35% to 52% at 1 month, respectively)^1,2^. About 65% of spontaneous ICH is caused by hypertension^3^. Patients who survive mechanical damage related to the hematoma effect may have additional deficits as a result of the brain injury.

Brain injuries following ICH include inflammation and thrombin activation. Thrombin is known to alter brain endothelial cell function, leading to disruption of the blood-brain barrier and subsequent brain edema formation^4^. Leukocyte infiltration also adds to brain injury through production of proinflammatory cytokines, chemokines, reactive oxygen species, and matrix metalloproteinases^5,6^.

Using an external ventricular drain (EVD) is one of the most common lifesaving procedures used in neurologic intensive care units. Several types of acute brain injuries could benefit from EVD insertion, including ICH with intraventricular extension, subarachnoid hemorrhage, and traumatic brain injury^7^.

The need to develop a reliable therapy to reduce brain injury following ICH and an appropriate and practical route to deliver the therapy is critical. Mesenchymal stem cells (MSCs) are multipotent cells with capacity to differentiate into multiple-cell lineages. MSC therapy appears to be a promising cell-based therapy, with proven safety and some efficacy in animal models and in clinical trials^8–10^. More importantly, MSCs have been shown to release cytokines or neurotrophic factors in a paracrine manner, affecting the damage niche surrounding target cells^11^. Moreover, MSCs are known to be immunosuppressive, and therefore, can ultimately control inflammatory response following ICH.

Several studies have demonstrated the effectiveness of MSCs in cerebral hypoxia-ischemia models^12–16^. However, to our knowledge, no study has evaluated MSC administration via EVD in a hemorrhagic stroke model or assessed the feasibility of using EVD catheter to deliver therapeutic MSC in a clinical trial. We assessed the safety and therapeutic effect of intraventricularly administered BM-MSCs in an ICH rat model.

## Materials and Methods

### Cell culture

Following Mayo Clinic guidelines for using biospecimens in research and after obtaining Institutional Review Board (IRB) exempt, excess deidentified bone marrow sample that was collected from consented healthy donor was used to generate MSCs. Mononuclear cells were isolated using Histopaque-1077 (Sigma-Aldrich Co. LLC.) density gradient protocols and used to culture to expand the MSCs. The MSCs were maintained in alpha minimum essential medium supplemented with 16.5% fetal bovine serum (designated as Complete Culture Medium) according to a previously reported method^17–19^.

### Animal model

**Figure 1:**
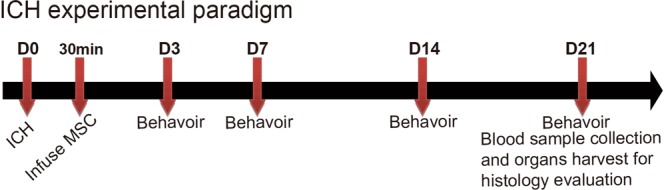
Rat Experimental Design. ICH surgery was performed on Day (D) 0. Twenty-four hours later, MSCs were intraventricularly infused. Starting from D3, rat behavior was assessed using forelimb use asymmetry test (cylinder test), performed at all time points (indicated by arrows). Blood samples and main organs were collected at D21 for further analysis.

All animal protocols were approved by the Mayo Clinic Institutional Animal Use and Care Committee. Twenty Sprague-Dawley rats (weight 250–275 g, Harlan Laboratories, Inc.) were used in this study. The ICH rat model was created as previously reported by Hua *et al*.^20^; the complete rat experimental paradigm is illustrated in Fig. 1. Surgery was performed on Day 0. 100 μl of autologous blood are infused into right basal ganglia at a rate of 10 μl/minute using a microinfusion pump with stereotaxic coordinators (0.2 mm anterior, 5.5 mm ventral and 3.5 mm right lateral to the bregma). MSCs were infused into the ventricle contralateral to the hematoma side by microinjection syringe which mimicking EVD drug delivery 30 minutes after autologous blood injection.

#### Intraventricular delivery

After ICH surgery, rats were randomly divided into 4 groups, with each group containing 4 rats. A separate control group that did not have surgery was used for comparison against the 4 active groups. One of the 4 ICH groups received phosphate-buffered saline (PBS), and the remaining 3 groups received intraventricular administration of MSCs at 3 different dose tiers (2 × 10^5^/kg, 5 × 10^5^/kg, and 1 × 10^6^/kg) in PBS (coordinates: 0.8 mm caudal, 4.8 mm ventral, and 1.5 mm left lateral to bregma).

### Forelimb use asymmetry test

Forelimb use asymmetry test was used to evaluate how well the rats’ forelimbs were performing with or without MSC treatment. ICH was induced on Day 0, and behavioral tests started on Days 3, 7, 14, and 21 post-surgery. Rats were placed in transparent cylinders (20 cm in diameter and 30 cm in height) and forelimb movements were counted and recorded. Behavior was scored as previously reported by Hua *et al*.^20^. Behavior was quantified by determining the total number of occasions when the unimpaired (ipsilateral) forelimb was used on the wall (I); the total number of occasions when the impaired forelimb (contralateral to the blood injection site) was used on the wall (C); and the total number of occasions when both forelimbs were used simultaneously (or nearly simultaneously during lateral side-stepping movements) on the wall (B). A single overall limb use asymmetry score was calculated as (I-C)/(I + C + B).

### BM-MSC toxicology assessments

Safety of intraventricular delivery of MSCs was assessed by observing the treated rat for changes in feeding habit, fur, overall behavior, and weight. Rat weight was documented throughout the experiment. Three weeks after MSC intraventricular administration, all rats were anesthetized with isoflurane; blood samples were collected through direct cardiac puncture and 500 µl of the blood was put into the BD Microtainer SST to isolate serum for comprehensive blood chemistry analysis. All remain blood sample was put into BD Vacutainer ACD Solution B tubes to isolate plasma for the cytokines analysis. The rats were perfused with PBS followed by 4% paraformaldehyde in PBS. They were then decapitated, whole brains were dissected, and heart, liver, lungs, spleen, testis, and kidneys were collected. All organs were kept in 4% paraformaldehyde for 24 hours, then washed with PBS twice and soaked in 70% ethanol. Paraffin embedding and sectioning, along with hematoxylin and eosin (H&E) and immunohistochemical staining were performed in the Mayo Clinic Cancer Center Histology Core facility. Cell toxicity evaluation on H&E staining slides were sent out to Vet Path Services, Inc. for an independent pathologist assessment.

### Immunohistochemistry and brain injury volume assessments

Slides from brain tissue were prepared and stained with anti-rat doublecortin (1:2000, Abcam, Ab18723, Sapphire Bioscience Pty Ltd) to evaluate therapy-induced neurogenesis. Human lamin immunohistochemical staining was used to detect human cells in the various rat tissues evaluated. Similar to above, evaluation was performed by an independent veterinary pathologist. H&E slides were scanned with ScanScope XT system (Aperio), and volume of injury was calculated using ImageScope software (Aperio).

### Cytokine assay

Blood samples were collected directly from heart puncture and centrifuged to collect serum for cytokine assays. Rat Cytokine Array/Chemokine Array 27 Plex (RD27) (Eve Technologies Corporation) was used to evaluate cytokine and growth factor expression. Eotaxin, EGF, fractalkine, IFN-γ, IL-1α, IL-1β, IL-2, IL-4, IL-5, IL-6, IL-10, IL-12(p70), IL-13, IL-17A, IL-18, IP-10, GRO/KC, TNF-α, G-CSF, GM-CSF, MCP-1, Leptin, LIX, MIP-1α, MIP-2, RANTES, and VEGF were targeted in this assay.

### Statistical analysis

All data are presented as mean (SD). Data were analyzed by analysis of variance or Mann-Whitney test. *P* values less than 0.05 were considered statistically significant.

### Ethical approval

All applicable international, national, and/or institutional guidelines for the care and use of animals were followed. This article does not contain any studies with human participants performed by any of the authors.

## Results

### Safety of intraventricular administration of BM-MSCs in ICH rat model

**Figure 2:**
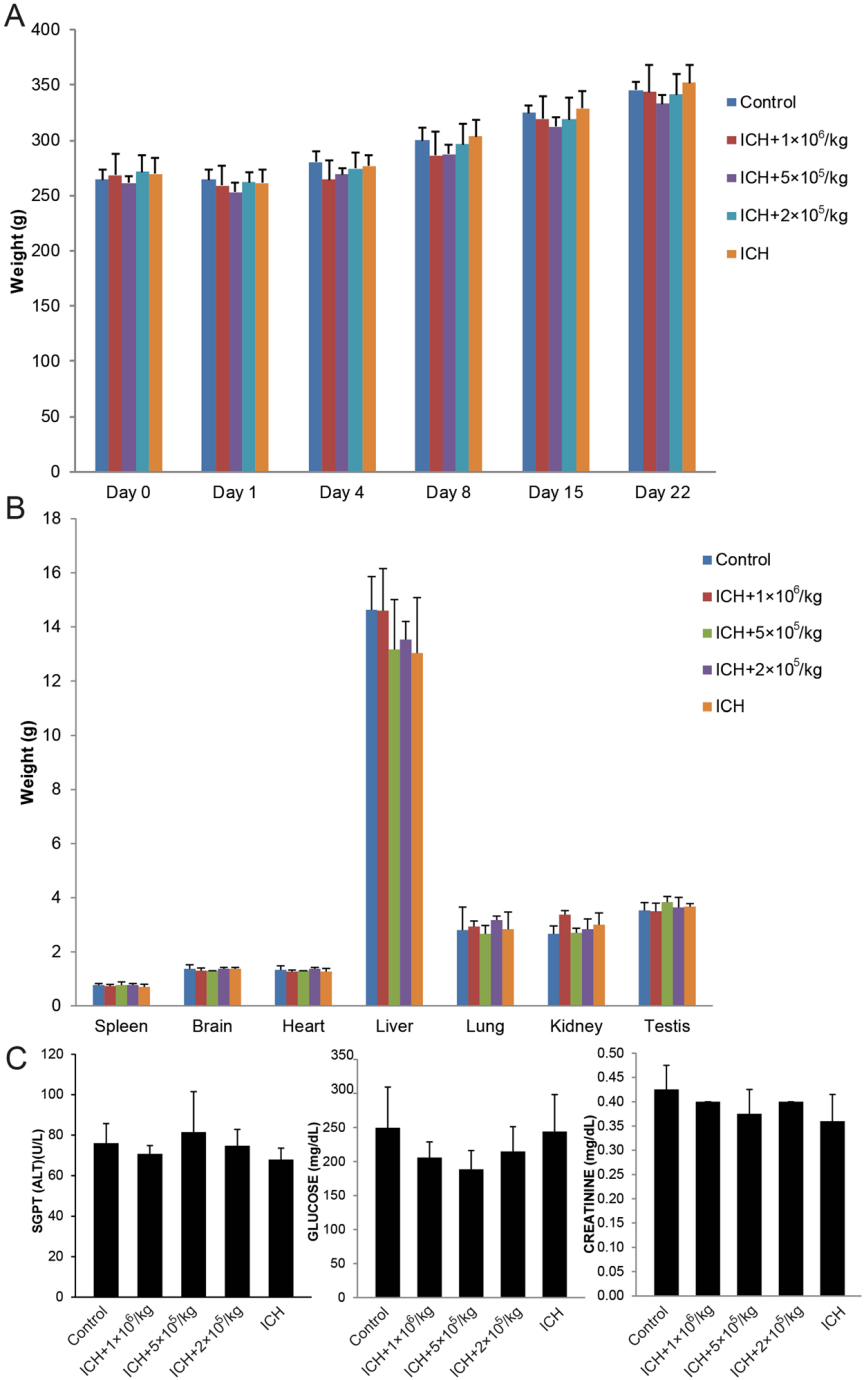
Intraventricular Administration of MSCs is feasible and safe. (**A**) Consecutive rat weight monitoring throughout the experiment showed no mean weight changes for 3 weeks. (**B**) Weight comparison of organs at the end of the experiment among all groups showed no notable changes in the mean rat organ weight. (**C**) Mean serum ALT, glucose and creatinine was not notably different among the rat groups compared with control group. ALT indicates alanine aminotransferase; SGPT, serum glutamic pyruvic transaminase.

Compared to the control group, the intraventricularly treated rats showed no notable deterioration of physical appearance or behavioral activities to suggest meningitis or major adverse event. There was no noteworthy weight difference among the 5 rat groups; all rats gradually gained weight overtime (Fig. 2A). At the end of the 21-day observation period, all rats were sacrificed and organs were dissected and weighed. Similarly, no major organ weight difference was notice among the groups (Fig. 2B). Liver and kidney functions were evaluated using alanine aminotransferase, glucose, and creatinine as biomarkers; no considerable difference was observed in the level of these markers (Fig. 2C). H&E slides from brain, heart, liver, lung, spleen, testis and kidney organ tissues showed no evidence of therapy-related cytopathic changes (data not shown).

### BM-MSC tracking and fate following intraventricular administration

We prepared immunohistochemistry slides stained with anti-human lamin A/C antibody to detect homing of the intraventricularly delivered cells to the organs evaluated. No lamin A/C-positive cells were observed in any of the organs. We validated our lamin A/C staining using a cytospin mixture of human BM-MSCs with rat cells and a tissue section of human renal cancer in a mouse kidney; the sensitivity of the assay was shown to be 1:10,000 (Supplementary Fig. 1).

### BM-MSCs reduced injury volume and enhanced neurogenesis following ICH

**Figure 3:**
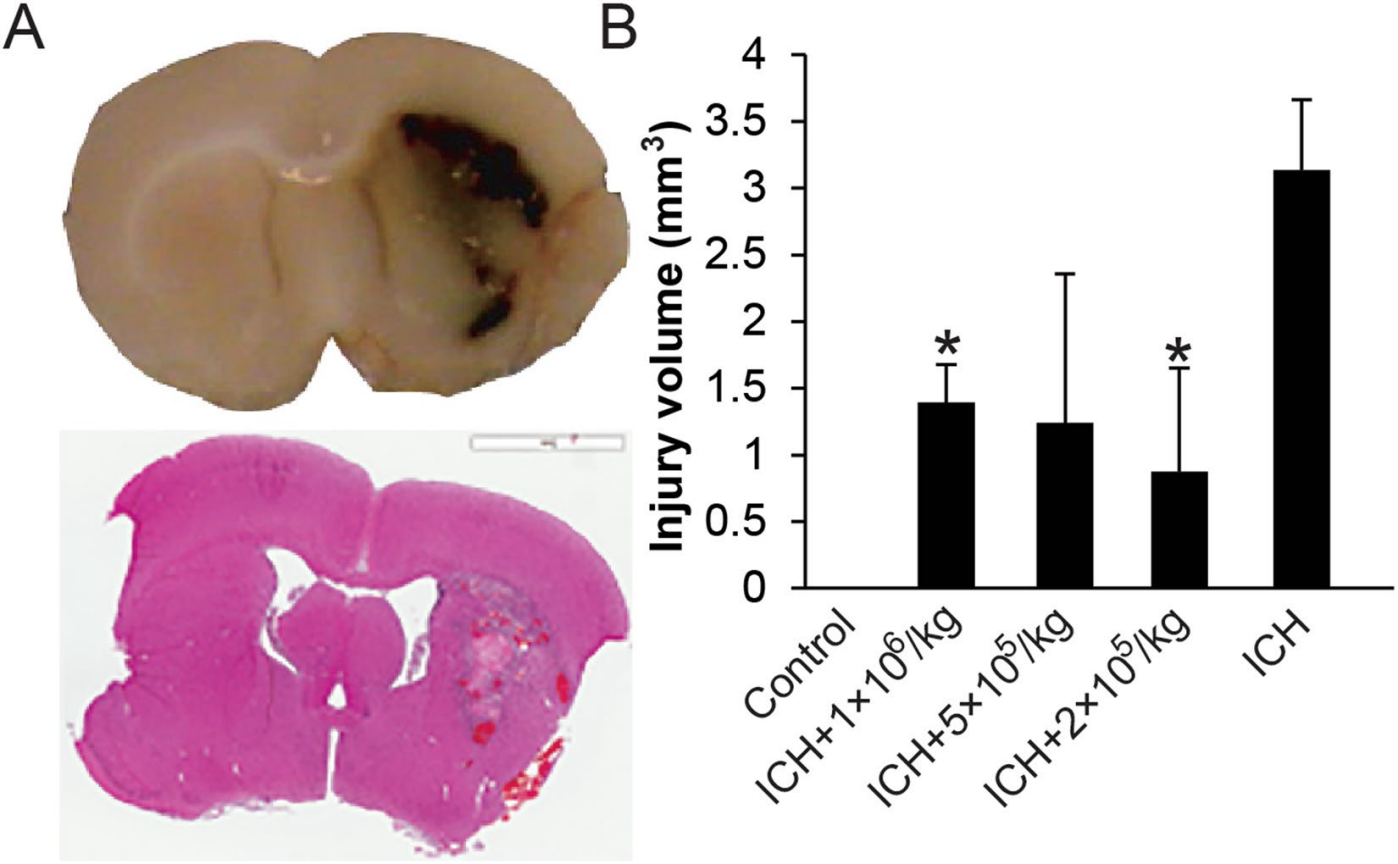
Brain Coronal Sections. Hematoxylin and eosin staining (**A**) and injury volume calculation (**B**) after intraventricular administration of MSCs were shown. Three weeks after infusion of 100 µL autologous blood, the damaged area was measured by ImageScope software. Means and standard deviations of the injury size were determined. The area significantly decreased in the 2 × 10^5^/kg and 1 × 10^6^/kg MSC-treated groups compared to the untreated ICH group. *Indicates *P* < 0.05 compared to ICH group.

**Figure 4:**
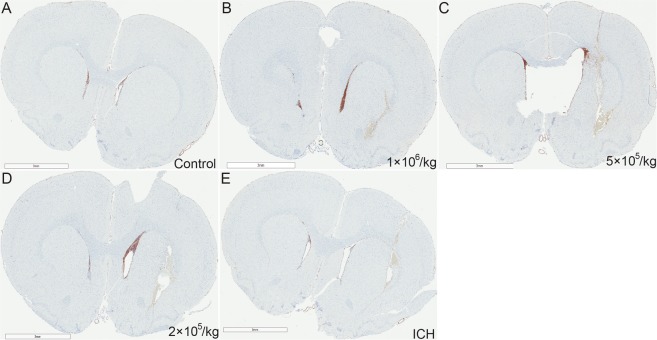
Intraventricular administration of MSCs Enhances Doublecortin Expression in Subventricular and Boundary Zones of the brain injury site. Several (>10) brain sections from each animal in each dose group were prepared. The figure represents the best section with the most evidence of neurogenesis from each group. (**A**) Representative image from control group with no ICH and MSC or PBS infusion. (**E**) Representative image from ICH control group that only received PBS. (**B**–**D**) Images from rats receiving ICH surgery with different dose of MSC infusion. Scale bar: 3 mm.

We evaluated the capacity of intraventricularly administered MSCs to alleviate brain injury and improve neurologic function. A typical brain injury is shown in Fig. 3A; MSC administration noticeably decreased mean injury volume after 3 weeks (Fig. 3B). All 3 MSC dosage groups demonstrated reduced mean injury volume, but only the 2 × 10^5^/kg and 1 × 10^6^/kg MSC groups showed statistically significant difference (about 75% and 50% reduction in injury volume, respectively) compared to control group (*P* < 0.05). To assess the capacity of MSCs to induce neurogenesis *in vivo*, we evaluated expression of neuronal precursor cell marker, doublecortin, expressed around the ICH injury site. Compared to the control group, MSC-treated rats showed more doublecortin expression, which was an indication of enhanced neurogenesis in the treated groups (Fig. 4).

### BM-MSC–modulated inflammatory response in ICH model

**Figure 5:**
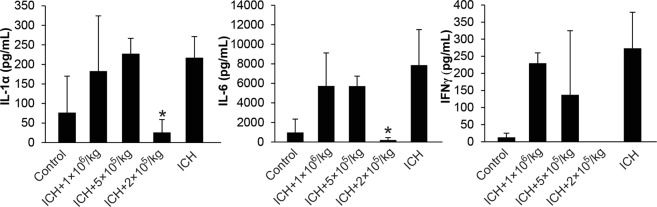
MSC Decreased Inflammatory Response in ICH Rat Model. Rat Cytokine Array/Chemokine Array 27 Plex (RD27) Assay was performed on rat serum samples collected 21 days after intraventricular administration of MSCs. We saw significant reduction of proinflammatory cytokines IL-1α, IL-6 and IFNγ. *Indicates *P* < 0.05 compared to ICH group.

Three weeks after ICH and MSC transplant, blood samples were collected, and serum samples were isolated. Rat Cytokine Array/Chemokine Array 27 Plex (RD27) was used to analyze inflammatory factor expression. Only the 2 × 10^5^/kg group showed significantly reduced mean expression levels of IL-1α, IL-6, and IFN-γ (Fig. 5). Therefore, intraventricular MSCs decreased inflammatory response by modulating cytokine expression.

### BM-MSC–induced functional recovery following ICH-induced brain injury

**Figure 6:**
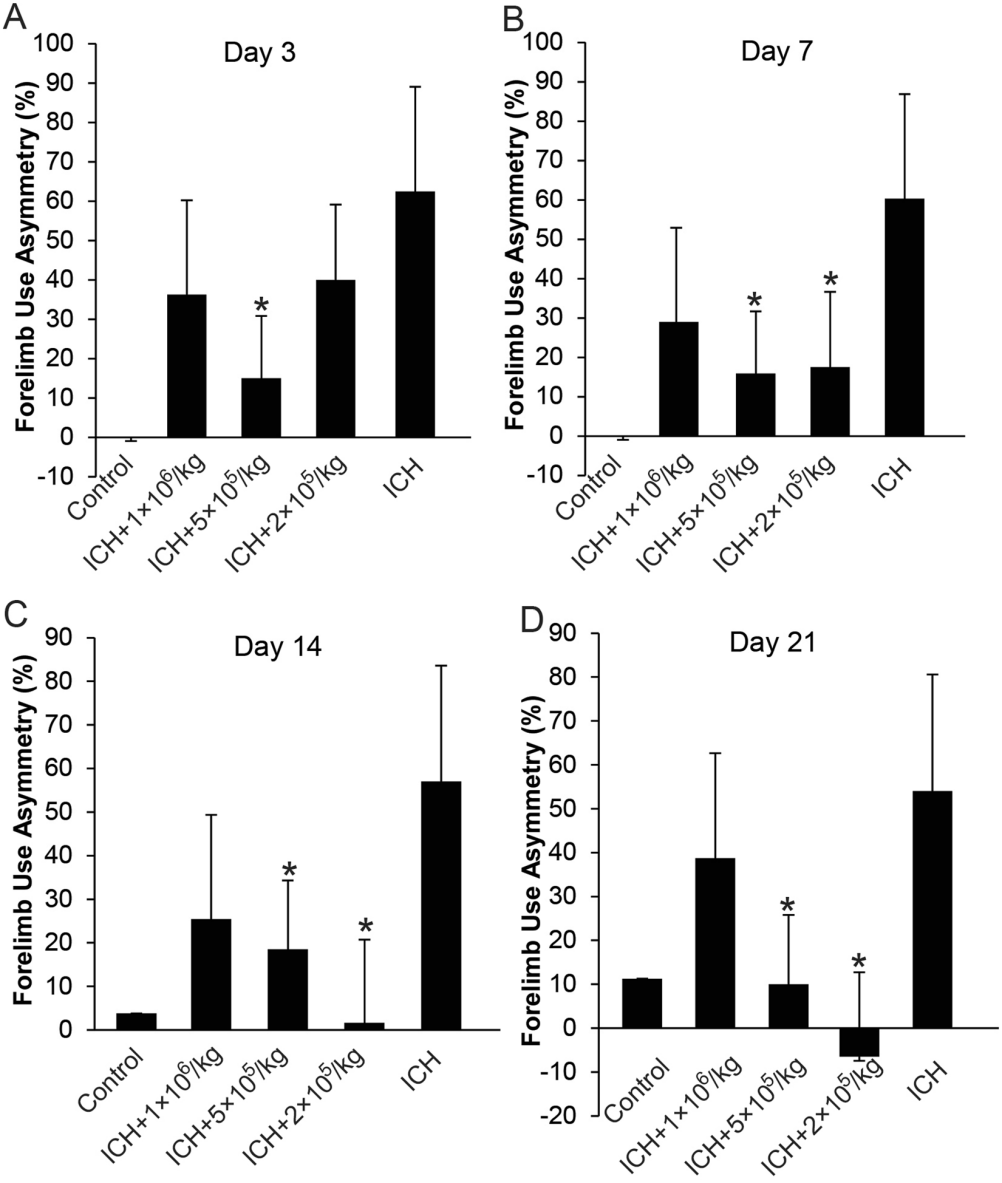
Behavior Analysis with Forelimb Use Asymmetry Test. (**A**) 3 days; (B) 7 days; (**C**). 14 days; and (**D**). 21 days after ICH surgery. Control group did not receive ICH surgery. ICH group received PBS only, while the remaining groups received different doses of intraventricularly administered MSCs. *Indicates *P* < 0.05 compared to ICH group.

To evaluate whether MSCs could facilitate recovery from ICH-induced neurologic deficits, forelimb use asymmetry tests were performed as outlined in Fig. 1. Starting from Day 3, the 5 × 10^5^/kg MSC group showed substantial improvement in limb function compare with the control group. Over the 3-week period, the 2 × 10^5^/kg MSC group demonstrated the best improvement in limb function (Fig. 6).

## Discussion

While ICH comprises only 10% to 15% of all strokes, the disease carries a mortality rate of up to 40% by 1 month. ICH is the only stroke type that lacks any US Food and Drug Administration-approved therapy that alters clinical outcomes^21^. Spontaneous ICH can extend into the cerebrospinal fluid cavities causing intraventricular hemorrhage (IVH), which leads to increased intracranial pressure (ICP), decreased cerebral perfusion pressure, and potentially global brain ischemia unless there is an intervention.

The use of EVD helps alleviate obstruction of cerebrospinal fluid flow or hydrocephalus, which if left untreated, causes raised ICP when ICH and IVH occur. An EVD is the standard method of both measuring ICP and draining cerebrospinal fluid; therefore, the procedure is both diagnostic for measuring and therapeutic in reducing ICP. We see EVD as a logical route for drug delivery or other novel therapeutics, such as MSCs. EVD also bypasses the drug blood-brain barrier challenges experienced with other routes of delivery, such as enteral or intravenous^7,22^. Our study showed that intraventricular delivery of MSCs in an ICH rat model was as safe as other delivery methods^23^. Consistent with previous reports^16,24–26^, our data demonstrated that MSCs were effective in reducing ICH-induced brain injury size and enhancing neurogenesis following ICH. MSCs also appear to play some anti-inflammatory role by substantially reducing expression levels of IL-1α, IL-6, and IFN-γ^27,28^. The scope of this study is narrowed to safety and efficacy of using EVD to deliver MSC in ICH model. The study was performed to generate preclinical data to support our clinical trial. So we did not include experiments to address mechanism of MSC induced neuroprotection and anti-inflammation in the study design. However, we had performed experiments just to address this question and our findings were reported in Huang *et al*.^29^. We believe MSC act by tropism through secretion of IL-6 and VEGF.

In most of the studies involving MSC therapy to treat ICH, the cells were delivered intravenously^24,26^, intrathecally (spinal canal subarachnoid space)^30^, or intranasally^31^. While intrathecal injections can get cells into the subarachnoid space, we felt an intraventricular route would translate better to human ICH with IVH. EVD catheters are often placed intraventricularly as part of clinical care, while subarachnoid catheters are not routinely placed for ICH with IVH. Also, a few studies have used intraventricular administration of MSCs, both preclinically and in human studies, to attenuate hypoxic-ischemic injury following traumatic brain injury^28,32,33^. To our knowledge, our study is the first to evaluate the use of EVD catheter to deliver MSCs into the cerebral ventricle to treat ICH-induced brain injury and inflammation in adults. The MSC treatment in this study was in the contralateral side because the injury site is very hostile to all cells including MSC due to ischemia and inflammation. Unpublished data from our lab show that direct transplantation of MSCs to the ipsilateral ventricle did not help with behavior improvement. Actually, on day 11, the rat group receiving treatment on ipsilateral, direct injection appeared to be as functionally compromised, in terms of forelimb asymmetry, as the untreated rat group (ICH only). Therefore, we designed our study to deliver the cells to the contralateral ventricle.

ICH management warrants immediate aggressive care to reduce the risk of medical adverse effects. Rapid diagnosis and attentive management of ICH patients is crucial because deterioration usually happens in the first few hours after disease onset. More than 20% of patients will have a decrease in Glasgow Coma Scale score of 2 or more points between the prehospital emergency medical services assessment and initial evaluation in the emergency department^34,35^. Therefore, timing of MSC therapy and an efficient means of delivery are critical for the therapy to be effective. Study suggests that MSCs are more likely to be effectual in an acute setting because a few inflammatory cytokines such IL-1, TNF-α, and INF-γ are known to activate MSCs and make them more potent^28^. Thus, use of an EVD catheter to deliver MSCs will ensure maximal therapeutic value in the setting of acute ICH. Our study showed intraventricular delivery of MSCs is as effective as other delivery methods in decreasing injury volume, reducing inflammation, facilitating neurogenesis, and improving function following ICH. Comparing the lifespan of rat with human and the therapeutic windows of human ICH, we have determined that 30 minutes is best therapeutic window for a 72 hour window for our clinical study.

It is not clear which method of MSC delivery will result in superior therapeutic value. Du *et al*.^36^ performed an animal experiment to compare different administration routes of adipose-derived stem cells in an ischemic rat model. Intraventricular, intra-arterial, and intravenous routes for implantation of adipose-derived stem cells have all shown neurologic improvement and reduction in infarct volume after ischemic stroke^36^. Based on our earlier experiments using intravenous delivery of MSC in the rat ICH model, we demonstrated 1 × 10^6^ cells/kg to have the best therapeutic efficacy. Unpublished data from our lab showed comparing Intravenous, Intrathecal and direct injection routes of MSC administration, direct injection demonstrated the least therapeutic effect. In general, intravenous delivery is generally preferred because it is technically easier and safer than other delivery methods. Further studies comparing multiple MSC delivery methods to the injury site are needed to determine the most efficacious approach, lowest effective cell dose, and above all, greatest safety.

We could not ascertain the fate of the intraventricularly delivered MSCs after 21 days post-infusion using human laminin A/C antibody. Previous reports using other delivery methods and a more sensitive method, such as magnetic resonance imaging/single-photon emission computed tomography/fluorescent tri-modal labelling, showed that 35% of intracerebrally injected MSCs migrate along the corpus callosum to the lesion area, while 90% of intravenously injected MSCs remain trapped in the lung at 14 days after MSC transplant^37^. Additional, time points that the cells were able to be detected were at 3 days, 1 week and 2 weeks. However, the detection frequency was very variable and inconsistent. A very recent study by Namioka *et al*. that involved intravenous infusion of GFP labeled MSC in ischemic stroke model reported MSC detection rate in the infarct site to be 7.4% on day 3, 1.3% on day 14 and 0.28% on day 28^38^. Another study by van Velthoven *et al*. reported no cells could be detected by day 70^39^. We were not able to detect intraventricularly injected MSCs in the brain beyond 21 days.

## Conclusion

Our study demonstrates the safety, feasibility and therapeutic efficacy of intraventricular administration of BM-MSC in an ICH rat model and further supports the idea of using an EVD catheter to deliver MSCs to treat ICH-induced injury and inflammation.

## Supplementary information

Supplementary information
