# Supplementary material for: Safety and Efficacy of Intraventricular Delivery of Bone Marrow-Derived Mesenchymal Stem Cells in Hemorrhagic Stroke Model
Source: Sci Rep. 2019 Apr 5;9:5674. doi: 10.1038/s41598-019-42182-1 (PMC6450980; doi:10.1038/s41598-019-42182-1)

Safety and Efficacy of Intraventricular Delivery of Bone Marrow-Derived Mesenchymal Stem Cells in Hemorrhagic Stroke Model

Peng Huang ^1^, William D. Freeman ^2,3,4^, Brandy H. Edenfield ^5^, Thomas G. Brott ^2^, James F. Meschia ^2^, Abba C. Zubair ^1*^

**Supplementary Figure 1** Anti-Lamin A/C Staining of Human Mesenchymal Stem Cell (MSC) mixed with Rat PC-12 Cell Lines. Human MSC and Rat PC-12 cell lines were mixed at different ratios then loaded on to PLOY-L-LYSINE-coated slides using cytospin technique. Anti-human lamin A/C monoclonal antibody was applied for immunohistochemical staining with hematoxylin costaining for nucleus. Positive staining was shown as nucleus localization. PC-12 cells are smaller than MSCs. Scale bar: 50 µm.

Supplementary figure 1


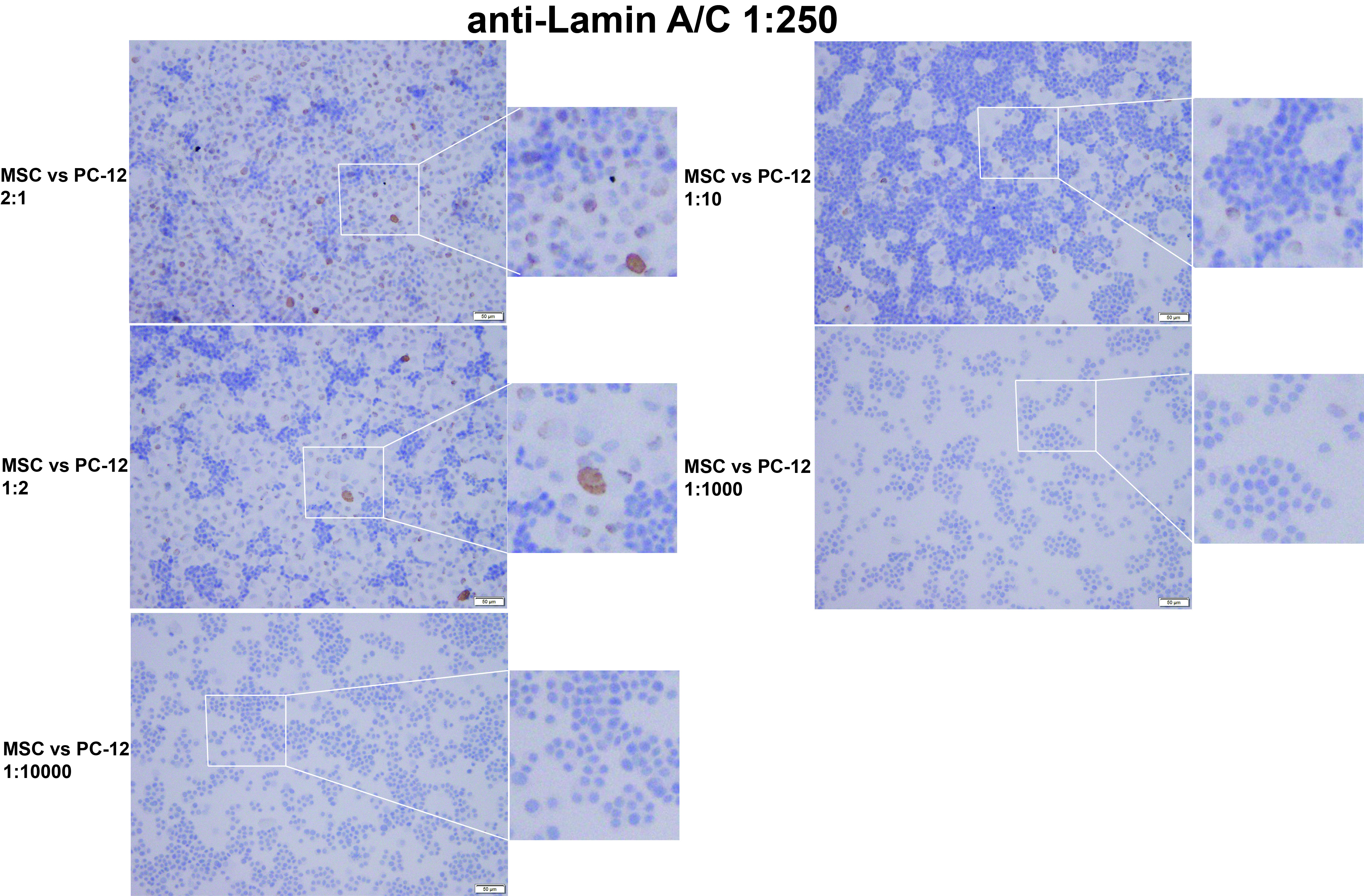


**Supplementary Figure 2** Brain coronal sections (HE staining and injury volume calculation) 1 week after infusion of 100µl autologous blood. Brain injury area was measured by image scope software. The area significantly decreased in 1x10^6^/kg MSC treated group compare to control group. Control group receives PBS only (* P<0.05 compared to control).

Supplementary figure 2


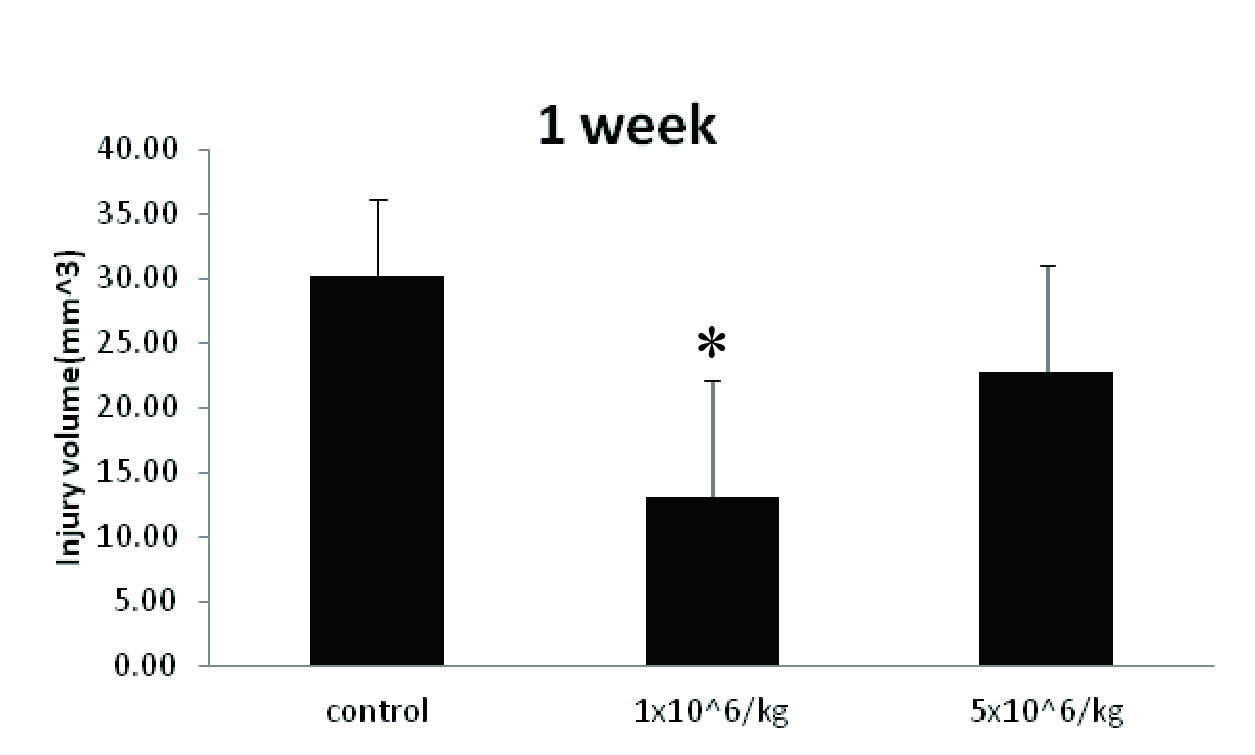

Supplement: Supplementary file 1 — Supplementary information [file 41598_2019_42182_MOESM1_ESM.docx]
